# On the Treatment of Pulmonary Consumption by Hygiene, Climate and Medicine

**Published:** 1867-01

**Authors:** J. Henry Bennett


### Miscellaneous.

### On the Treatment of Pulmonary Consumption by Hygiene, Climate and Medicine.

BY J. HENRY BENNETT, M. D.

In my younger days this fatal and cruel error was carried to an insane extent by many medical practitioners, as it still is in most parts of the continent, and especially in Germany. The windows were often hermetically shut, and paper pasted over the chinks. The doors were made double, and one always shut before the other was opened. The healthy friends of a patient considered it a penance and a trial to have to remain in the polluted atmosphere "necessary" for the miserable sufferer, and often paid for their devotion by the loss of their own lives. On the other hand, the wretched patients suffered from constant suffocation as well as from a steady aggravation of the symptoms of the disease. This suffocation, the mere result of want of pure air, was called dyspnœa, and treated by opiates and sedatives instead of by opening the windows.

All my consumptive patients, whatever the stage of the disease, live night and day in a pure atmosphere, obtained by allowing a current of air to pass constantly through the room, either by a more or less open window and open fireplace, or by a door opening on a well-ventilated staircase if the weather does not admit of the window being even slightly open. Rational, reasonable ventilation is not encompassed, however, without trouble and discrimination for human beings any more than for plants, although it is to be accomplished. Since I have been an invalid I have devoted much time and study to horticulture, and have had former convictions as to the necessity of efficient ventilation thereby confirmed. Plants under glass, too crowded and not well ventilated, soon sicken, wither, and die. To well ventilate them, enough and not too much, require constant trouble, attention, and good sense on the part of the gardener; in a word, exercise of both judgment and discrimination.

Consumptive patients bear ventilating perfectly well, as well as healthy people, day and night. They neither get pleurisy nor pneumonia, nor are their coughs aggravated by breathing pure, cool, atmospheric air night and day; whereas all these evils pursue those who are shut up, as are the numerous continental patients whom I see in consultation with their own doctors every winter at Mentone. Moreover, suffocation, medically called dyspnœa, is all but unknown, even in the latter stages of the disease, to those who are allowed plenty of fresh, cool air. It soon comes on, however, if the window is shut and the room becomes close. These persons, accustomed to free ventilation, *will* have more air; indeed, I often stand aghast at the amount of ventilation such patients, previously freed by me from groundless fears, insist on having. All who have damaged lung-tissue, unless accustomed by long habit to a close atmosphere, feel more or less oppression in a confined atmosphere, owing to the diminished field of their respiration. A concert room, a theater, a close chamber at night, bring on dyspnœa all but immediately. This I have learnt from personal experience, and thus most fully can I sympathize with my patients.

I fully admit, however, that free ventilation without dangerous draughts is difficult to attain, and that it is much more easily and safely accomplished in a southern than in a northern climate.

Before I leave this subject I would draw attention to the physiological fact that the lungs are made to breathe cold as well as warm air—indeed, air of any temperature from zero to 100 ° Fahr., just as the face is made to bear exposure to the external atmosphere. How could the lungs be protected?—if they required protection, which they do not. Domestic animals that live out in the open air winter and summer are freer from colds than those that live in warm stables; and men who are much exposed, and constantly breathe air at low temperature, are less liable to colds and influenza thaa those who live constantly in warm rooms. All who have horses are aware that to keep a stable warm is the surest way for the inmates to suffer from constant colds.

I may mention two facts that aptly illustrate the evils of defective ventilation. Some years ago I was riding in the Highlands of Scotland with a local proprietor, when we came upon a village of well-built stone houses with slate roofs, which strongly contrasted with the miserable shanties or hovels generally met with. On my complimenting him on his rebuilt village, he told me that he had acted for the best in erecting these good weather-proof houses for his tenants, but that, singular to relate, they had proved more unhealthy than the miserable dwellings which their occupants previously inhabited. Fever and other diseases had proved rife among the latter. On examination, I found that the windows were fastened, and never opened; and I have no doubt that their comparative unhealthiness was in reality owing to their being quite weather-tight, and consequently unventilated. In the miserable hovels they previously inhabited, if the rain of heaven come in, so did pure air.

The other fact is narrated by Prof. Hind in a recent interesting work on Labrador. Consumption appears to be all but unknown to the natives living wild in the fastnesses of this desolate region, in tents made of spruce branches imperfectly lined with skins, and more or less open on all sides to the external air; although they are exposed to famine and every species of hardship. But when these same natives come down to the St. Lawrence to take a part in the fisheries, occupy well-built houses, and, being well paid, live in comparative luxury, most of them in the course of a year or two become consumptive and die miserably. I am fully impressed with the idea that the development of the disease under these circumstances is the result of their living in close houses in a vitiated atmosphere, as it no doubt is in our own towns.

Attention to the functions of the skin is, I consider, next in importance to attention to food and air—that is, to digestion and to respiratory nutrition. The skin has very important eliminatory functions to perform. It is by excretion through its pores that the economy partly throws off the effete or used-up carbonaceous and nitrogenous elements of the system. This is illustrated by the strong odor of the cutaneous secretion when not washed off. Moreover, the skin and the lungs seem partly to replace each other in this work of excretory purification. In warm, summer weather, the skin and liver act freely, and the lungs and kidneys are comparatively at rest. In the cold, damp weather of winter, the pores of the skin are closed, and it rests, the lungs and kidneys taking up the excretory process. Thence probably the feverish colds of damp, cold weather. The blood is poisoned with the elements that the closed pores of the skin should have eliminated, which occasions the fever; whilst the lungs often succumb to the increased duties they have to perform, and inflammatory affections supervene. Whatever the explanation, the fact is certain, and it is now well established that the best mode of preserving the respiratory organs from winter colds is to keep the pores of the skin open by the use of cold or tepid water, combined with friction; or, in other words, to keep the cutaneous excretions up to their normal standard.

Acting in accordance with this view, I make all my consumptive patients, whatever their condition, if they have the strength, use a sponge-bath at a temperature of from 64° to 68° daily, and with the greatest possible benefit. I neither have to contend with hemorrhage or chills, nor with aggravation of the cough, but quite the contrary. The cold sponge-bath produces in nearly every instance a feeling of indescribable comfort, and lowers the pulse. The contact of the cold water may accelerate the expectoration of the muco-pus collected during the night in the bronchial tubes, but that never alarms when it is explained that such a result is naturally to be expected. I myself derived the greatest possible comfort and benefit from cold sponging in summer in the open air on the banks of a Scotch loch, the waters of which were at 60°; and that when I was very ill, pulse 100, and skin hot and feverish. This gave me a confidence I have never lost, and of which I have never had reason to repent.

The question of exercise is an important one, and one that requires discussion and elucidation. I would say at once that, from personal experience and observation, I believe it is a great mistake for consumptive patients to take much active exercise. Every winter I see some such patients walk themselves to death. They have been told by their medical attendants at home to take exercise, and they do so, thinking that what gave them an appetite and did them good when well will do so now when they are ill; but they merely walk themselves into their graves. The disease from which they are suffering is one of debility. The strength of former days has gone out of the youth and of the man, altho' perhaps he knows it not. Or the strength he has is fictitious, unreal strength, the result of a febrile condition, of a state of morbid nervous excitement. So he walks up hill and down dale, loses his appetite, can not eat, becomes "bilious," is dosed for liver, and the disease progresses rapidly. Every winter, toward January or February, some invalids consult me who have up to that time taken their case in their own hands, and have thus walked from breakfast to dinner, with the healthy, in order to gain strength. But they have lost it indeed—have become paler and thinner; and when I see them, I find that they have lost ground, that the disease has gained upon them since they arrived in the autumn, and that they are decidedly worse—all from over-exercise.

The sound rule for a consumptive patient is to take passive exercise, not active; to ride in an open carriage; to be rowed in a boat; to sit and lie hours in the open air; to live with windows open, but never to incur great muscular exertion. The amount of vital power in such cases is small. If it is too freely expended in exercise, there is not enough left for normal digestion; food is imperfectly assimilated, nutrition is defective, and the disease progresses.

A singular, but explicable fact is, that during the existence of active disease, when tubercles are forming and softening, very often no lassitude is felt on exertion. But when the disease is arrested, and a curative process has been set up, extreme debility and lassitude may be experienced and complained of, lasting for months, or even years. I felt this lassitude for five years. The explanation is simple. As I have already stated, in active disease there may be a false, feverish strength, like that of the delirious patient whom it takes half a dozen men to hold. In the curative stage, the false strength is gone; the real condition of the patient comes to light, as it does with the delirious patient when the delirium is gone, and he can scarcely lift his hand from the bed.

The social and mental hygienic condition favorable to the treatment of consumption may be summed up in a few words. Rest, repose, the absence of the ordinary duties, cares, harass, and worries of life. To obtain these is difficult in the social medium in which the disease has appeared. Therefore, the duties and obligations of life should be surrendered for a time, if possible; modified, diminished, if not. Those, however, have the best chance of arresting the progress of disease who can escape from the social medium in which it appeared. To do this it is always necessary to make great sacrifices—sacrifices which many can not make. But those who can must remember that the struggle is one, not merely for a higher or lower stage of health, but for life itself.

*Medicinal Treatment of Phthisis.*—I have now reached the most difficult part of my subject, one that skill affords great room for difference of opinion, even if the premises contained in the previous papers are admitted. It would be vain to endeavor to reconcile the conflicting views which reign in the profession respecting the therapeutics of phthisis, so I shall confine myself to a statement of the conclusions at which I have arrived from my own personal experience.

As that experience has increased, I have gradually arrived at the conviction that there is no medicinal panacea for pulmonary tuberculosis, any more than for any other form of tuberculosis. There is no one remedy, in my opinion—no one drug that can act as an antidote to this morbid diathesis; neither cod-liver oil, nor iodine, nor iron, nor the preparations of phosphorus, nor any other pharmaceutical agent. Those who believe that there is such an antidote appear to me to ignore the very nature of the disease not to be aware that it is merely the local evidence or symptoms of exhausted vitality, of general vital decay, a mode of death manifesting itself as to the result of worn-out organic power.

Such a condition is not to be remedied by physic, but mainly by physiology, with physic as an adjuvant, a handmaiden. It is only by removing all the causes that are depressing life, contrary to the healthy development of the functions of life, and by placing the sufferer in the most favorable hygienic conditions for the development of his organization, that we can hope to arrest or cure such a disease. Here, again, horticultare has been of use to me. If a plant is failing because it is of a bad stock, or because it is placed in conditions of air, moisture, sun, shade, or soil, unfavorable to its habits and nature, it is not by adding this manure or that to the soil in which it grows that it can be restored to health. All such efforts are vain. Its nature and habits must be studied, and then the conditions favorable to its healthy development in every respect must be adopted. Once this is done, a favorable change must take place, provided its vitality be not already too depressed, or provided disease has not advanced too far to admit of recovery. At the same time, well chosen manures, the addition of a necessary element deficient in the soil, will materially help the horticulturist.

So it is with physic in phthisis. Although no mere drug can give new life to a decaying organization, can arrest and cure a disease in itself a mere symptom of such decay, an enlightened use of medicinal agencies may do much to aid improved hygienic conditions, in rousing and restoring vitality, and in arresting the progress of disease. There are many stumbling blocks in the path of consumptive invalids, many conditions of disordered functional activity, which render the most hygienic treatment nugatory, and which physic has the power to modify and remove. Such are disordered conditions of stomach, liver, and intestines; morbid states of innervation, cerebral and spinal; uterine, vesical, rectal complications, functional or local, all of which are more or less under the influence of medicine.

To meet these and other complications we have numerous and valuable medicinal agents at our call; mineral acids, alkalies, vegetable bitters, sedatives, narcotics, alteratives, astringents, all of which in turn do good service in the hands of the experienced physician. There are few stages or conditions of the disease in which such a practitioner does not find an important indication, something to do medicinally, by which nature and hygiene may be assisted in their operations. I am a firm believer in physic, and seldom or ever leave my consumptive patients entirely to nature. I firmly believe that I can help them by the application of rational therapeutics, and try to do so. When myself a consumptive invalid, for many years I was always doing something in the way of medicinal treatment; and have the decided conviction, right or wrong, that I increased my chance of recovering by thus meeting the varying phases of my own case.

Having laid down on a broad basis the principles which should, in my opinion, regulate the treatment of phthisis, I have a few words to say on some of the therapeutical agents which stand highest in the professional mind, preëminent among which is cod-liver oil.

Prof. Bennett, of Edinburgh, first introduced this agent to the profession in Great Britain, in a work written, *ex professio*, on the subject in 1842. He had found it extensively given in Germany; and in this work communicated to his countrymen the experience of his German friends, as also his own. A few years later, Dr. C. J. B. Williams, our most eminent and enlightened thoracic pathologist, gave cod-liver oil the sanction of his great experience. From that time its influence in favorably modifying nutrition, and in arresting tuberculosis in the lungs, has been universally acknowledged; so that now it has become the great remedy for consumption, and that most deservedly. Some of our American brethren state that, since its general use in phthisis, the mortality from the disease has sensibly diminished, and, as a result, that the general death-rate is lower.

The question naturally presents itself, if cod-liver oil undoubtedly exercises a beneficial and even curative influence on pulmonary tuberculosis, why and how does it produce this effect? Chemical analysis of fish-oil does not give a clue, for the amount of iodine and bromine discovered is so infinitesimal that we can hardly admit that theirs is the potent influence; especially when we find that, administered alone in these or even in larger doses, the therapeutical effect is not produced. To discover the clue we must fall back upon physiology.

It is now generally admitted by physiologists that fatty substances, if not absolutely essential to digestion and nutrition, exercises a most beneficial influence over these processes; indeed, nature appears to have placed facts within the reach of man all over the world, and to have implanted instinctive craving for them in mankind. In northern climates, the natives consume largely fish oils alone or with their food; in temperate climates butter and meat fats take their place; while in sub-tropical regions, vegetable oils, such as olive oil, form an important element of the food; even in the tropics there is the palm oil, and gee, or butter, to satisfy the absolute want of fatty substances. From physiological requirements to those of the morbid condition of nutrition which constitute tuberculosis there is only a step, which observation has made. It has been long remarked that in these morbid conditions of the human economy a larger amount of fatty nutritive elements than is necessary for food becomes a means of restoring nutrition to a more healthy state, and thus that these become positively therapeutical agencies.

If this view of the action of cod-liver oil is the correct one—if in giving it we are merely ministering, in an exaggerated degree for therapeutical purposes, to a natural health requirement, any fatty substance would have the same result. Within certain limits I believe that such is the case; cream, fat meat, vegetable oils, bacon, butter, etc., all answer the physiological condition; and I invariably give them, if possible, when the patient's stomach can not bear the fish oil. But I also believe with the rest of the profession, that the fish oil is the best, is the easiest digested and assimilated, and is the fat to which the stomach gets the soonest reconciled, and which it can take the longest. I myself took an ounce and a half a day for five years without intermission, at last with pleasure, and always with benefit to the digestive processes. A medical friend of mine, well known to the profession, who has, like myself, saved his life by the combined influence of hygiene, climate, and physic, could never take cod-liver oil; "but then," says he, "I took fabulous quantities of butter with my meals."

It is a known and admitted fact that the greater number of those who now recover from phthisis are persons who have taken cod liver oil. This fact certainly redounds to the credit of the remedy, but it must be remembered that those only can take it in whom the digestive organs are in a sound condition natarally, or in whom they have been restored to a sound condition by proper medical treatment. Women in whom uterine disease sympathetically produces nausea and sickness—those who are suffering from chronic dyspepsia, or from chronic liver or kidney disease—generally speaking, can not take the fish oil; it nauseates them, makes them sick, and destroys their appetite, as often as do all fatty substances. Thus, the recovery of those who can, and do, take cod-liver oil may be not so much because they take it as that their digestive system is sound, and that they can take and digest fat and plenty of good nourishing food besides. On the other hand, those who can not take the oil, and die, may die not so much because they do not take the oil remedy, as that their digestive system is bad, and can not be restored to a healthy state, so as to admit of the food-cure.

The undoubted improvement of the majority of the consumptive patients who can take cod-liver oil or other fats, in health, in strength, and in condition, has received additional and most valuable explanation and confirmation from some recent interesting physiological experiments. These experiments have been made during the last year by Dr. E. Smith, the Rev. Prof. Haughton, Dr. Frankland, and Professors Fick and Wislicenn, of Zurich, with a view to arrive at a clearer notion than we had before respecting the strength or power shown or spent by animated beings. They have been carried on under the influence of modern views respecting the correlation of physical forces, and the doctrine of the conservation of force, and of the equivalency of heat and mechanical force. The generally-received physiological idea of nutrition is, that nitrogenous or albuminous food, by the process of assimilation, is transformed into muscle and force; whereas carbonaceous, fatty, amylaceous food is burnt, and generates animal heat. The experimentalists whom I have quoted appear to have satisfactorily established that the production of the muscular power spent by animals and man is not so much to be attributed to the assimulation of nitrogenous food as to the slow combustion of carbonaceous food. According to this theory the formation of animal heat by the combustion of carbon is attended with the development of "force," of which the muscles may possibly be only the instruments, not the producers.

This view may be familiarly explained by the steam engine; the latter, in burning coal, does not only produce heat, but power—the power that drags the train along. In a more obscure but equally evident manner the slow combustion of food in the processes of nutrition is attended with the development, not only of heat, but of power—force. If the above views are correct, it would follow, singular as the statement appears, that more power or strength is to be got out of fat than out of meat or muscular tissue; and this really appears to be the case. Tyrolese chamois hunters find that they can endure greater fatigue on beef fat than on the same weight of lean meat, and, accordingly, when about to absent themselves for several days in the mountains, they take beef fat with them instead of meat. (See *Intellectual Observer*, July, 1866.)

Thus is explained the craving of mankind for fatty food, and for carbonaceous food generally. Thus is illustrated the generally acknowledged physiological principle that man is omniverous, and is also explained the strength of the rice eating Hindoo and of the potato eating Irishman. A rational dietary is evidently the one in which nitrogenous and carbonaceous food are mingled in due proportion.

Lastly, we may safely conclude that fats are not "bilious"—bile producers, as popularly believed; but that the inability to digest them is merely an evidence of defective, or of weak and easily disturbed, digestive powers. The great majority of those whose digestive system is in good order digest fats with the greatest ease, and that in large quantities. The dislike so often shown to fat by persons in good health is often merely a result of education—of mothers most foolishly and erroneously picking out the fat from their children's food in early life "as unwholesome and bilious."

Iodine has a great reputation in the treatment of other forms of tuberculosis, and especially scrofula, which may be said to be almost the same disease. I presume this reputation is a deserved one; but as iodine is always administered conjointly with a generous dietary, and with hygienic treatment generally, it is very difficult to form an estimate as to its real value. In pulmonary phthisis it certainly does not appear to me to exercise much influence; and as it is apt to disorder the stomach and to interfere with the appetite, I now seldom give it internally. I constantly, however, use it externally, over the diseased regions of the lungs, as a counter-irritant, and to promote absorption of adhesions.

Once it is admitted that the treatment of phthisis ought to be sthenic—invigorating, not antiphlogistic or debilitating—iron and and its preparations naturally present themselves to the mind. I have often administered them, and I believe with benefit, in the stage of convalescence; of retrogression, when tubercle is no longer deposited, but in process of absorption or cretefaction; and when the period of debility and lassitude supervenes. I have also given them during the acute stage, but I think without beneficial result. Indeed, at that stage they appear to me, like iodine, often to disorder the stomach, and to interfere with the appetite and digestion. When I observe this under any modification, I at once stop the remedy, firmly believing that food is of more value than physic if the choice is between the two. It is a remarkable fact, that no physician writing on chalybeate or iron waters recommend them for active phthisis; indeed, the opinion that they are dangerous appears to prevail.

Preparations of phosphorus, and especially the hypophosphites of soda and lime, were introduced by Dr. Churchill some ten or twelve years ago as a positive remedy for pulmonary phthisis. This is still Dr. Churchill's belief; and I, who have known him from early life, am convinced that he is sincere—that he really believes that he has found an antidote, a remedy for pulmonary consumption. The subject has of course much occupied my thoughts, and during the last seven years I have administered the drug to a large proportion of those whom I have attended.

Were I only to quote the successful cases that I have had under my care, the cases in which the tubercular disease has been arrested and even cured, I could furnish Dr. Churchill with many instances of cure, myself included, which have apparently taken place under the influence of the hypophosphites, as they were long or constantly administered. But, on the other hand, I have quite as many, perhaps more, cases of death to narrate in patients whose condition admitted of recovery from the extent of the disease, and who perseveringly took the hypophosphites from the beginning to the end.

Were the preparations of phosphorus given really an antidote to the disease, and the cause of the recovery in the first class of cases, they ought to have cured many of the latter, for they were all placed under the same hygienic and social conditions. The scrutiny and comparison of these cases of success and non-success, however, have left in my mind the conviction that the different results obtained are to be explained by considerations of general pathology, by the type of the disease, the constitution of the patient, the conditions under which it was generated, and that the patients were not taking a remedy that had the power to control antecedents and conditions unfavorable to recovery. It is worthy also of remark, that I have always administered either Dr. Churchill's own preparations, or salts furnished by his own manufacturer, so that there is a certainty as to the genuineness of the drugs used.

Although not admitting that phosphorus and its preparations are an antidote to pulmonary phthisis, for I have seen too many cases of failure to be able to admit it, I believe that they constitute a valuable medicine in asthenic disease, and especially in tuberculosis. Their administration, also, is quite rational physiologically, and I may say also agriculturally. Phosphate of lime is one of the principal elements of our economy. It forms the bones, and is found in our tissues, and especially in the brain and nervous tissues. It is sound physiology and pathology to give freely to the animal system in food, or as physic, the elements of which the system is composed. If it is right to do so in health, it is equally so in disease. In tuberculosis, observation shows that it is right to increase the usual amount of fat given in the system, and my observation seems to show me that it is equally right to increase the amount of phosphates. Phosphorus is only contained in limited amount in our food, although it exists in so large a proportion in our system. Its administration in a disease of debility may, in my opinion, be compared to manuring an exhausted field. If corn is grown several years in succession in the same soil, the crop at last fails for want of phosphate of lime, which is necessary to form the grain. Add bone dust, or phosphate of lime, and the corn comes up vigorously, and the grain forms healthily and well. It is in this sense that I give the preparations of phosphorus, and that I myself took them for five years.

The above views must have gained greater credence and weight with the profession than is generally admitted, for I seldom am consulted by a new patient at Mentone, each successive winter, without finding that he or she has been taking phosphorus in some shape or other, and that when their prescriptions are signed by the heads of the profession. Indeed, although I do not think Dr. Churchill is warranted in claiming for phosphorus the position which he gives it as a "remedy," for pulmonary consumption, I consider that the thanks of the profession are due to him for directing our attention to a valuable therapeutic agent in this dread disease.

In a sthenic, or strengthening treatment, such as I describe, in the curable stage of the disease, opiates can have but little place. What availeth it to allay irritation, to quiet the cough, and procure sleep, if thereby the appetite for food is destroyed, as is usually the case the day after an opiate night draught? Is it not better that the patient should have a moderate amount of distress and discomfort and eat, if eating is life and fasting death? In the latter stages of disease, when all hope of recovery is gone, and it is merely a question of soothing the last stage of life, then opiates become an inestimable blessing in judicious hands. There are, however, other sedatives—prussic acid, hyoscyamus, belladonna, conium—from which much ease may be obtained in the earlier stage of the disease, when there is still hope of recovery.

As to expectorants, I can not say that I have much faith or reliance in them. If muco-pus is abundantly secreted, it is better away, and nature expels it by the natural and then easy process of coughing. When secretion diminishes, as it does as the disease diminishes, and the patient coughs spasmodically to get rid of a sticky, tenacious secretion, which causes tickling and irritation, I

do not see what good is done by loosening it, as the term is, by squills, etc., even if they have the power to do so, which I doubt. The real remedy is an effort of strong will on the part of the patient to repress coughing until the natural action of the bronchial villi has pushed the muco-pus into the larynx, whence it can easily be expelled. My attention was first drawn by Professor Bennett to the fact that the dry, irritating cough for which expectorants are generally ordered is often merely the result of positive improvement, and is best left alone.

The local inflammations of pulmonary tissues around softening tubercles, the local pleurisies which are the result of tubercular deposits reaching the surface of the lung, are no doubt benefitted by counter-irritation—painting the chest with caustic iodine, by croton oil liniments, by small blisters, etc.; but I question whether much good is done by issues. Indeed, I think the pain and annoyance often counterbalance all the good done, and that the remedy is out of all measure with the benefit obtainable by its employment. The inflammation can only be radically cured by the natural subsidence of the causes of internal irritation, which the counter-irritation of the issue does not in the least control.

A volume might be written on the treatment of phthisis according to these views; but I propose limiting myself to the above brief general exposition, leaving my readers to fill it up themselves. I have now only to devote a page or two to the consideration of the "results of treatment," and I shall have accomplished my self-imposed task.—*Cincinnati Journal of Medicine.*

